# A detectable change in the air-sea CO_**2**_ flux estimate from sailboat measurements

**DOI:** 10.1038/s41598-024-53159-0

**Published:** 2024-02-09

**Authors:** Jacqueline Behncke, Peter Landschützer, Toste Tanhua

**Affiliations:** 1 Max Planck Institute for Meteorology and International Max Planck Research School on Earth System Modelling, Bundesstrasse 53, 20146 Hamburg, Germany; 2 Flanders Marine Institute (VLIZ), Jacobsenstraat 1, 8400 Ostend, Belgium; 3 Max Planck Institute for Meteorology, Bundesstrasse 53, 20146 Hamburg, Germany; 4 GEOMAR Helmholtz Centre for Ocean Research, Wichhofstrasse 1-3, 24148 Kiel, Germany

**Keywords:** Biogeochemistry, Carbon cycle, Climate sciences, Ocean sciences, Ocean sciences, Marine chemistry, Physical oceanography

## Abstract

The sailboat *Seaexplorer* collected underway sea surface partial pressure of CO_2_ (pCO_2_) data for 129 days (2018–2021), including an Antarctic circumnavigation. By comparing ensembles of data-driven air-sea CO_2_ fluxes computed with and without sailboat data and applying a detection algorithm, we show that these sailboat observations significantly increase the regional carbon uptake in the North Atlantic and decrease it in the Southern Ocean. While compensating changes in both basins limit the global effect, the Southern Ocean–particularly frontal regions (40°S–60°S) during summertime—exhibited the largest air-sea CO_2_ flux changes, averaging 20% of the regional mean. Assessing the sensitivity of the air-sea CO_2_ flux to measurement uncertainty, the results stay robust within the expected random measurement uncertainty (± 5 μatm) but remain undetectable with a measurement offset of 5 µatm. We thus conclude that sailboats fill essential measurement gaps in remote ocean regions.

## Introduction

The ocean plays a critical role in regulating Earth's climate by absorbing more than a quarter of anthropogenically emitted carbon dioxide (CO_2_) from the atmosphere on an annual basis^1–3^. However, climate change has already started to alter the carbon uptake capacity of the ocean^1,4^, thus monitoring the sea surface CO_2_ content is crucial for understanding the Earth system as a whole. Although there has been a significant community effort resulting in the collection and synthesis of sea surface CO_2_ observations^5,6^ in recent decades, and methods to upscale the existing measurements^7–12^ we find a significant difference between hemispheres. While the Northern Hemisphere has been regularly sampled in the recent past being the result of the community-driven measurement efforts resulting from the Ship Of Opportunity (SOOP) program^6,13^, key regions in the ocean carbon and heat uptake such as the Southern Ocean remain undersampled^5,14,15^. The resulting uncertainty in air-sea CO_2_ fluxes is problematic^16,17^ and limits our ability to resolve and interpret observed and modelled variations in the carbon sink^18–20^. This is concerning as the Southern Ocean alone is estimated to be responsible for 40% of the marine anthropogenic CO_2_ and 75% of the marine excess heat uptake^7,21^.

New techniques, including new sensors on biogeochemical floats, have started to address this observational gap, but their indirect measurements of pCO_2_—calculated from pH and salinity measurements—remain uncertain^22–24^. Additionally, Antarctic operations from Saildrones^25^ have contributed to filling the measurement gaps and are suggested to improve the air-sea CO_2_ flux estimates^26^, however, thus far no continuous measurement program exists. Given the limitations of the existing observational network and the moderate success of gap-filling methods in further improving pCO_2_ estimates^16,17^, it is essential to explore new opportunities to fill observational gaps.

Here we show that a novel observing platform is capable of improving our estimates of the air-sea CO_2_ exchange. Since 2018, the high-performance IMOCA class 60 sailboat “Seaexplorer-Yacht Club de Monaco “(until 2019 “Malizia”) has collected pCO_2_ observations (hereinafter: *Seaexplorer* data) while competing for 129 days in round-the-world racing events, including an Antarctic circumnavigation race from November 2020 to January 2021^27^. We show that the use of a single platform (“Seaexplorer-Yacht Club de Monaco”), and the participation in a single race in the Southern Ocean has a measurable effect on data-driven air-sea CO_2_ flux estimates. This impact persists even when considering its expected measurement uncertainty of ± 5 μatm^28^. Thus sailboats have the potential to complement and improve the existing observing system. Nevertheless, we further illustrate that high standard measurements are crucial in detecting changes in the air-sea flux and that measurement biases still pose a challenge for detecting improvements in the air-sea CO_2_ flux estimates.

## Results

### Global effect of adding sailboat pCO_2_ data

**Figure 1:**
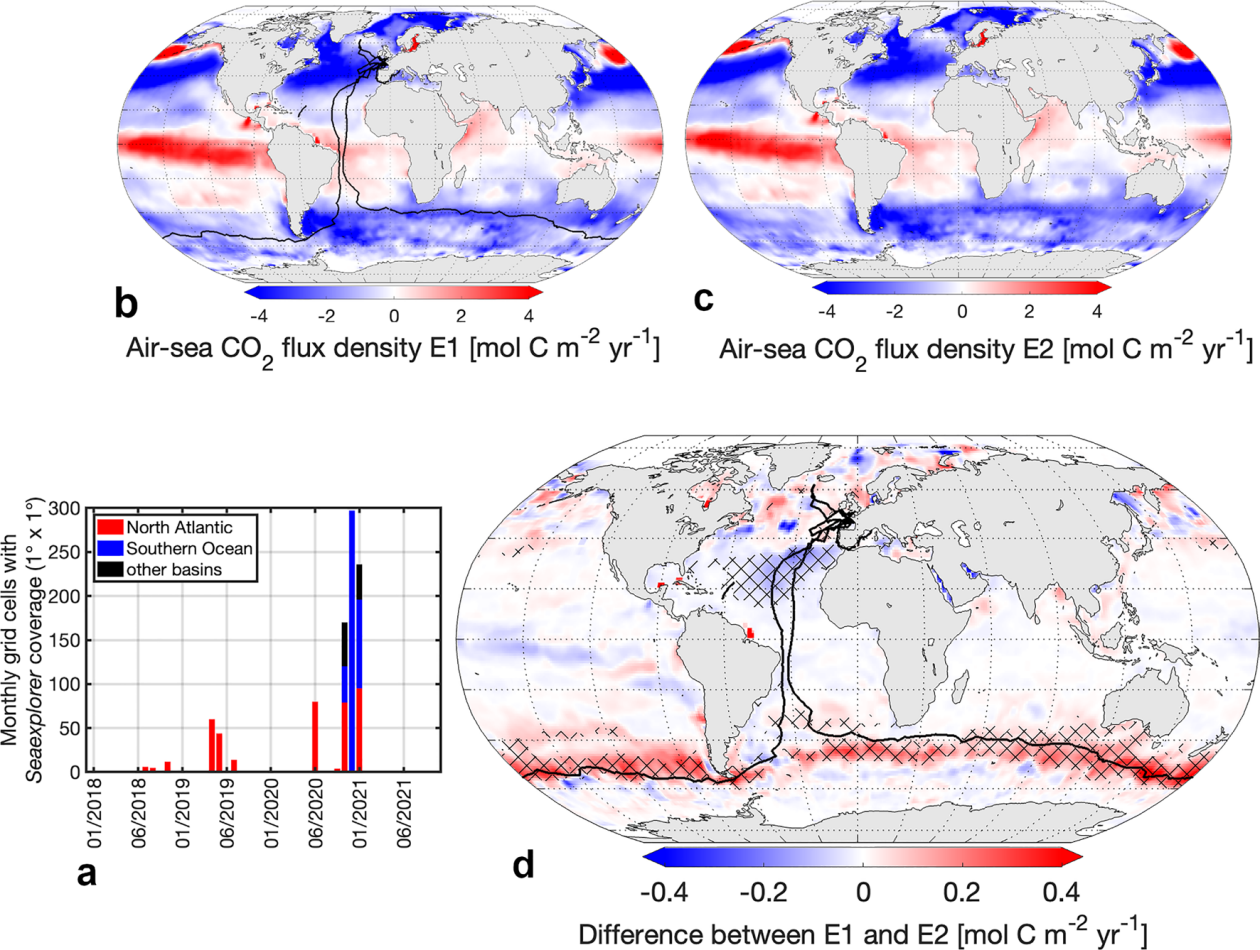
(**a**) Timeseries of *Seaexplorer* data availability per basin and b-d) air-sea CO_2_ fluxes in ensemble 1 (E1) and 2 (E2) and their difference averaged over Nov 2020–Jan 2021. (**b**) Air-sea CO_2_ flux in E1 (based on SOCATv2022 including Seaexplorer data). (**c**) Air-sea CO_2_ flux in E2 (based on SOCATv2022 excluding Seaexplorer data). Positive = carbon outgassing, negative = carbon uptake. (**d**) Difference between E1 and E2. Hatching indicates significant differences. Blue indicates increased carbon uptake due to the addition of Seaexplorer data, red indicates reduced carbon uptake due to the addition of Seaexplorer data. Black lines in (**b**,**d**) represent sailboat tracks from 2018 to 2021. Figures generated using a mapping package for MATLAB^32^.

Figure 1a,b show the air-sea CO_2_ fluxes calculated based on the upscaling of all available pCO_2_ measurements including (ensemble 1 = E1) and excluding *Seaexplorer* data (ensemble 2 = E2). The ensembles were generated using SOM-FFN, a 2-step neural network method^29^—see “Methods”—regularly used in the Global Carbon Budget^1^ and the recent IPCC assessment^30^. The significant impact of adding all underway pCO_2_ observations from the sailboat on the air-sea CO_2_ flux from November 2020 through January 2021 is further illustrated in Fig. 1c. We chose this time period from November 2020 to January 2021 as it showed the largest flux impact by adding sailboat data, which is related to the circumnavigation race where Seaexplorer participated (see black lines in Fig. 1 and in Supplementary Fig. 1). Interestingly, significant differences between E1 and E2 in the North Atlantic (largely negative shown in blue: E1 < E2) and the Southern Ocean (largely positive shown in red: E1 > E2) in the air-sea CO_2_ fluxes are opposing each other (Fig. 1), resulting in an insignificant change when integrated globally (i.e. an annual flux difference in 2021 from − 2.55 to − 2.51 ± 0.4 Pg C yr^−1^^1^), which has also been suggested by^27^.

Considering that both the North Atlantic and the Southern Ocean are predominantly carbon sinks from 2018 onwards, the addition of *Seaexplorer* data reveals increased carbon uptake in the North Atlantic and reduced uptake in the Southern Ocean (Fig. 1 and Supplementary Fig. 1) similar to previous findings^22^.

**Figure 2:**
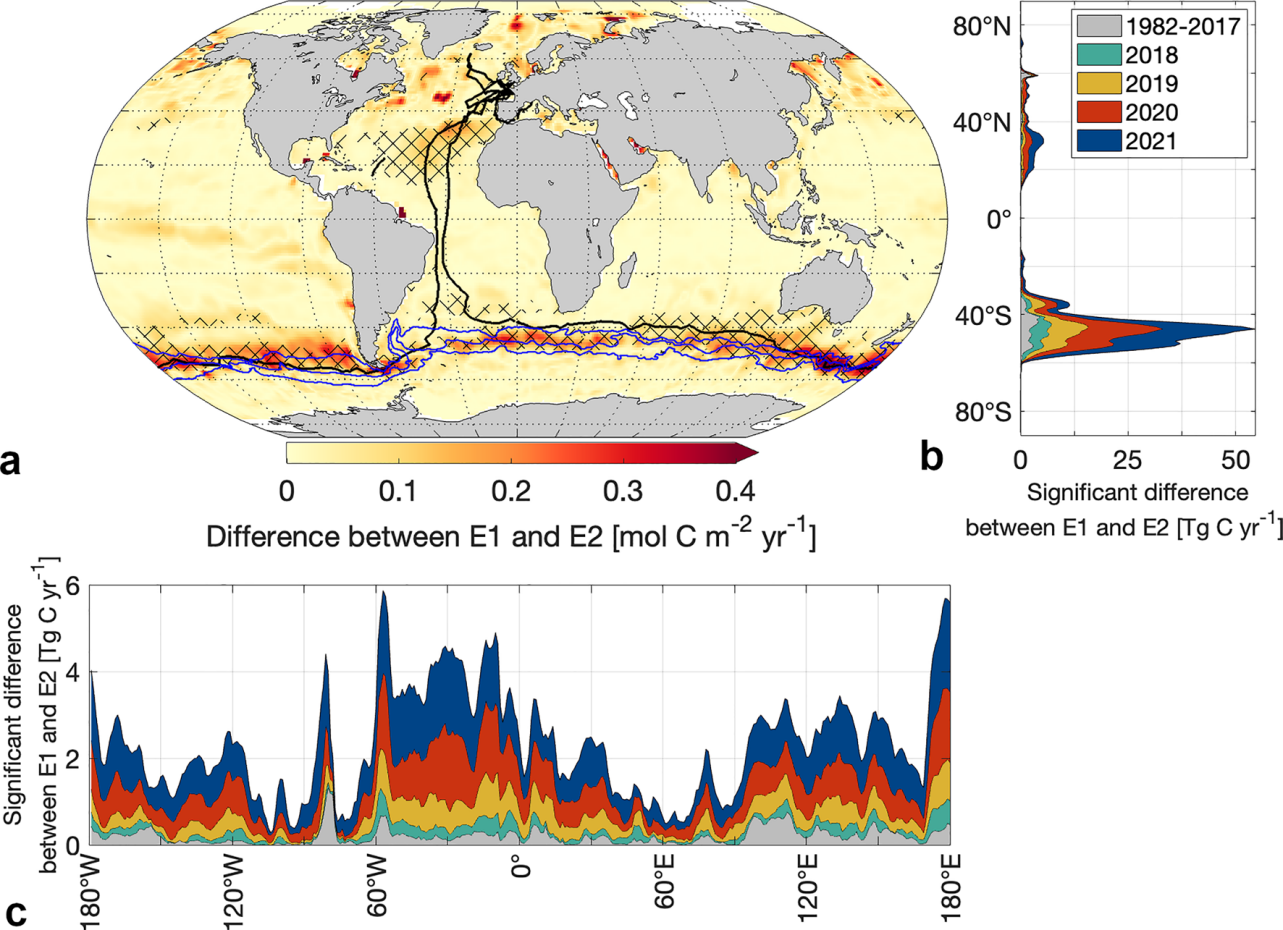
The absolute magnitude of differences between the air-sea CO_2_ flux E1 (based on *SOCATv2*022 including *Seaexplorer* data) and the air-sea CO_2_ flux E2 (based on SOCATv2022 excluding *Seaexplorer* data). (**a**) Map shows the absolute magnitude of differences between carbon flux estimates averaged over Nov 2020–Jan 2021. Hatching indicates significance. Black lines represent sailboat tracks from 2018 to 2021. Blue lines from north to south: Northern Boundary, Subantarctic Front, Polar Front. Figure generated using a mapping package for MATLAB^32^. (**b**,**c**) Significant differences between air-sea CO_2_ flux estimates per year and (**b**) latitude and (**c**) longitude.

Differences in the flux estimates are visible across all ocean regions even away from the sailboat tracks. The neural network's ability to estimate changes in air-sea CO_2_ flux distant from the sailboat tracks originates from its methodology, combining clustering and regression. This process involves assimilating data from observations made in distant yet biogeochemically comparable ocean regions. However, in many regions, these differences fall within the noise of the method^31^ (see “Methods”) and are thus not detected as significant changes (hatches in Figs. 1 and 2). This is most visible in the high-latitude ocean regions and is likely due to the poor constraint of the air-sea CO_2_ flux estimate in highly heterogeneous and sparsely observed regions^16,17^. Focusing on the detectable changes, irrespective of the background fluxes, the absolute magnitude of the difference between flux estimates provides a better insight (Fig. 2).

### Impact of adding sailboat pCO_2_ data in the Southern Ocean

In less frequently monitored regions such as the Southern Ocean, even adding Southern Ocean CO_2_ measurements from a single track results in a significant difference between E1 and E2 (Fig. 2a)—acknowledging a possible influence of sailboat observations from other oceanic regions that were equally excluded.

**Figure 3:**
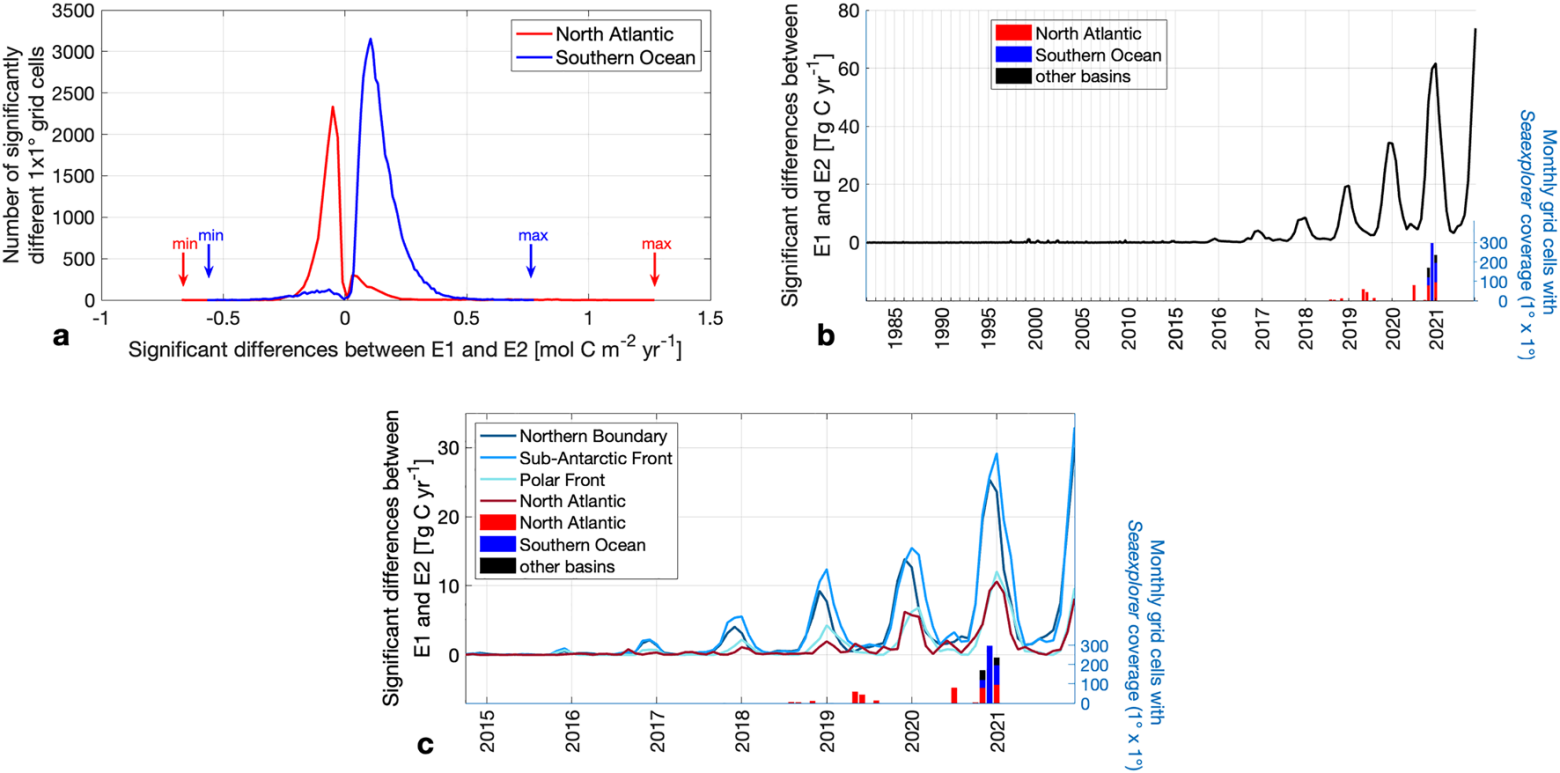
Magnitude of significant differences between the air-sea CO_2_ flux E1 (based on SOCATv2022 including *Seaexplorer* data) and the air-sea CO_2_ flux E2 (based on SOCATv2022 excluding *Seaexplorer* data). (**a**) Histogram of the magnitude of significant flux differences in the Southern Ocean and the North Atlantic. (**b**,**c**) Time series of the magnitude of significant differences between carbon flux estimates (based on SOCATv2022 with and without Seaexplorer data) as well as the Seaexplorer data availability per basin (**b**) on a global scale and (**c**) on regional scales.

This aligns with previous findings based on synthetic data^26^ demonstrating that few additional pCO_2_ sampling by Saildrone would potentially improve the air-sea CO_2_ flux reconstructions most in the Southern Ocean (south of 35°S). The reconstructions of our air-sea CO_2_ flux differ most significantly between 40°S and 60°S and with maximum differences of 0.77 mol C m^−2^ yr^−1^, reflecting the rate of carbon exchange between the atmosphere and the ocean per unit area, in the time period from 1982 to 2021 in the Southern Ocean (Figs. 2b and 3a). Overall, the absolute air-sea CO_2_ fluxes significantly differed on average by 0.15 mol C m^−2^ yr^−1^ in the Southern Ocean (Supplementary Fig. 2), which is roughly 20% of the regional mean flux density, thus leaving a significant imprint on the regional flux.

The impact of including the *Seaexplorer* data in the air-sea CO_2_ flux calculations is the largest within the vicinity of the Subantarctic Front (2-degree grid cells or approximately 200 km radius) closely followed by the Northern Boundary (Figs. 2a and 3c). Although the sailboat did not cross the Polar Front, significant differences emerge in its vicinity (Figs. 2a and 3c) due to the extrapolation of the data using the neural network algorithm. This pattern coincides with the coverage of the *Seaexplorer* data, as the region along the Subantarctic Front contained most *Seaexplorer* data with an overall 11% of the area covered by sailboat tracks when binned into a 1 × 1 degree grid, followed by 9% along the Northern Boundary, and 2% in the vicinity of the Polar Front.

Regionally, we find the largest differences during the Antarctic circumnavigation race between Nov 2020–Jan 2021 exceeding 0.4 mol C m^−2^ yr^−1^ in the interfrontal region south of Tasmania and New Zealand (Figs. 1 and 2a). Although the region south of Tasmania and New Zealand is relatively well-observed for the Southern Hemisphere^5^, the data availability close to frontal zones is insufficient considering the variability within this region^33^. The frontal zones are characterized by enhanced vertical mixing and high biological productivity. In fact, the pCO_2_ signal measured by Seaexplorer-Yacht Club de Monaco there is oversaturated and distinctly higher than the surrounding area^27^. Our results demonstrate the high potential of sailboat pCO_2_ data in improving the air-sea CO_2_ flux estimate in frontal regions.

Although our results confirm the finding that regional differences in the air-sea CO_2_ flux are attributed to the frontal zones in the Southern Ocean, the previously proposed changes south of the Polar Front^27^ probably stem from noise in the methodology and not from a detectable signal. This underscores the need for signal-to-noise detection methods as presented here, or alternatively, the use of synthetic data experiments using large ensembles^16,26^ when comparing different air-sea CO_2_ flux estimates from neural networks.

### Impact of adding sailboat pCO_2_ data in the North Atlantic

Compared to the Southern Ocean, individual races in the North Atlantic are less impactful (Figs. 2 and 3c), largely owing to the already denser observing network in place where the addition of a single measurement track does not cause large significant changes in the already robust reconstruction. Nevertheless, we still observe that sailboat pCO_2_ measurements have a significant regional impact since Seaexplorer data covers a total of 7% of the North Atlantic area (when binned into 1 × 1° pixels), in comparison to only 3% of the Southern Ocean area.

The air-sea CO_2_ fluxes significantly differed regionally peaking at 1.26 mol C m^−2^ yr^−1^ in the North Atlantic between 1982 and 2021 (Fig. 3a), which is thus higher than the maximum flux difference of 0.77 mol C m^−2^ yr^−1^ in the Southern Ocean. However, the mean difference of 0.08 mol C m^−2^ yr^−1^ in the North Atlantic is substantially smaller than observed in the Southern Ocean (0.15 mol C m^−2^ yr^−1^) (Supplementary Fig. 2), considering the historic coverage of the SOOP program. In recent years however, we also find a reduction in North Atlantic measurements (www.socat.info;^5^), thus even in the better observed North Atlantic the sailboat data might gain more importance.

### Temporal evolution

Comparing the flux reconstructions E1 and E2 over time, we see the greatest impact of adding *Seaexplorer* data from 2018 to 2021 in the air-sea CO_2_ flux estimates in the latter years of the time series (Fig. 3). About 91% of the significant differences between E1 and E2 occurred between 2018 and 2021, which is when the sailboat pCO_2_ observations were measured. The pCO_2_ data collected by “Seaexplorer-Yacht Club de Monaco” affects the air-sea CO_2_ flux estimate only up to ca. 5 years prior to the Antarctic circumnavigation race. This is not immediately obvious, since the applied method extrapolates information both in space and time. It learns from all available observations when clustering the ocean into biogeochemical provinces and estimating the missing pCO_2_ values by using previously established relationships between already available pCO_2_ and other environmental variables within each province. However, a similar observation, where differences become smaller as we look further back in time, has been made when BGC Argo data were added^22^. This is explained by trend variables (i.e. atmospheric xCO_2_) used in the method^29^ limiting the potential of the method when extrapolating the missing pCO_2_ values into the past^22^. As a consequence, we expect that a longer time series is required to change the interannual to decadal air-sea CO_2_ flux trends. Nevertheless, with upcoming races announced (round-the-globe racing events taking place every other year) and with the increasing willingness among skippers to contribute with observations, we see a long term potential to increase pCO_2_ data in remote ocean regions to overcome this limitation.

The addition of *Seaexplorer* data has the highest impact on austral summer, whereas it has little to no impact on austral wintertime fluxes (Fig. 3b,c), mirroring the seasonal availability of data and illustrating the weak connectivity between seasons identified in our neural network. Therefore, sailboat measurements—unlike Saildrone campaigns^15,16^—currently are unable to bridge the wintertime discrepancy between float-based and ship-based flux estimates^22^. Even though sailboat pCO_2_ data have limited added value during harsh winter conditions in the Southern Ocean where no sailboat racing events take place, we show that sailboats support the existing observing system of Argo floats^22,23,34^, Saildrones^25,35^, moorings, drifting buoys, and wave gliders.

### Sensitivity of air-sea CO_2_ flux to measurement uncertainty

**Figure 4:**
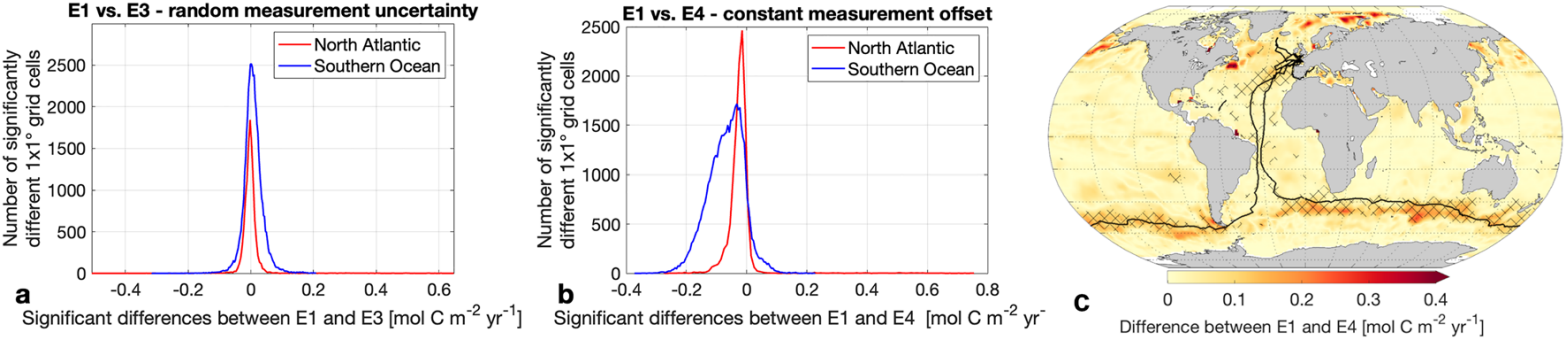
Sensitivity of air-sea CO_2_ flux to measurement uncertainty. (**a**,**b**) Histogram of significant differences between flux estimate E1 (based on SOCATv2022 with original *Seaexplorer* data) and E3 and E4 (based on SOCATv2022 with modified *Seaexplorer* data) in the Southern Ocean and the North Atlantic. (**a**) E3 modification = addition of random measurement uncertainty, (**b**) E4 modification = addition of constant measurement offset. (**c**) Map shows the absolute magnitude of differences between the original flux estimate E1 and the E4 flux estimate including a sailboat measurement offset averaged over Nov 2020–Jan 2021. Hatching indicates significance. Black lines represent sailboat tracks. Figures generated using a mapping package for MATLAB^32^.

Finally, we also tested whether potential measurement uncertainties or even measurement bias has an effect on the air-sea CO_2_ flux estimate. We considered a random measurement uncertainty of ± 5 μatm (ensemble E3) and a constant measurement offset of 5 μatm (ensemble E4) (Fig. 4) as the data set quality flag assigned by SOCAT is 5 μatm. Figure 4a illustrates that a random measurement uncertainty of ± 5 μatm does not affect the air-sea CO_2_ flux at a basin-wide level in the North Atlantic and the Southern Ocean, as the mean difference (E1–E3) is near zero for both basins (Fig. 4a). However, locally the air-sea CO_2_ flux can be significantly impacted by up to 0.65 mol C m^−2^ yr^−1^ in the North Atlantic during individual months and up to 0.32 mol C m^−2^ yr^−1^ in the Southern Ocean (Fig. 4a). This highlights the importance of accounting for measurement uncertainty when investigating high-frequency and small spatial scale fluxes which will become increasingly important as we move towards marine carbon accounting, marine carbon dioxide removal and national carbon stocktake efforts^36,37^.

We also explore potential limitations of the system and imperfect calibration over long offshore racing events by testing a constant measurement offset of 5 μatm. This causes a global bias up to 0.06 Pg C yr^−1^ (E1–E4) in 2021 (Supplementary Table 1). On the one hand this is only roughly 2.5% of the global annual uptake, showing the rather small sensitivity of the air-sea CO_2_ flux, to biases in a single platform. However it is larger than the global flux change from adding *Seaexplorer* data of 0.04 Pg C yr^−1^ (Supplementary Table 1). Thus, our comparison highlights that flux changes from measurements from 129 days at sea remain undetectable if the measurement system does not follow the highest standards, supporting the need for a CO_2_ reference network^38^. However, while important globally, the constant measurement biases are still smaller at basin scale. The mean absolute difference in the air-sea CO_2_ flux attributed to the offset is 0.03 mol C m^−2^ yr^−1^ in the North Atlantic and only 0.07 mol C m^−2^ yr^−1^ in the Southern Ocean (Fig. 4b), which is smaller than the mean differences caused by adding Seaexplorer data. Particularly in the North Atlantic, the flux estimate proves to be more robust towards a potential measurement offset than the Southern Ocean flux estimate. This robustness is again attributed to the already denser observations from different platforms. This however also indicates the need for cross-calibration of measurements in remote regions, as measurement biases have a larger impact there. Locally, the air-sea CO_2_ flux densities (based on E1-E4) significantly differed up to 0.76 mol C m^−2^ yr^−1^ in the North Atlantic during individual months and up to 0.38 mol C m^−2^ yr^−1^ in the Southern Ocean. The significant differences occur in proximity to the sailboat tracks and peak between 40°S and 60°S (Fig. 4c). Equipping more sailboats with a pCO_2_ measurement device during the round-the-world races would help to reduce the impact of potential measurement uncertainties and increase the accuracy of our flux estimate. Considering that many studies thus far do not include or assess the impact of measurement uncertainty in their pCO_2_ observations^7,39^, we hereby show the importance of measurement uncertainty analyses at a regional scale to provide a more accurate estimate of high-frequency fluxes.

## Discussion

We quantify the impact of underway pCO_2_ data from sailboats on the air-sea CO_2_ exchange by comparing the air-sea CO_2_ flux estimates based on pCO_2_ measurements of the SOCAT database—with and without the *Seaexplorer* data. We show that measuring pCO_2_ underway, and in particular during round-the-world sailing events, improved air-sea CO_2_ flux reconstruction at regional scales, particularly in under-sampled regions like the Southern Ocean. However, we find that also in the more densely observed North Atlantic, significant flux density changes occur locally. The flux reconstructions differ the most between 40°S and 60°S, particularly within 200 km of the Subantarctic Front during austral summertime, where the largest disagreement in air-sea CO_2_ flux reconstructions exist^40,41^. While the addition of Seaexplorer data regionally increases the estimated carbon sink in the North Atlantic, it reduces the carbon sink in the Southern Ocean similar to previous studies^22^. Even though sailboat data cannot help to close the winter discrepancy between float-based and ship-based flux estimates^22^, due to the seasonal sampling bias, it supports the existing observational platforms (www.socat.info). Utilizing this data, particularly in combination with various other platforms, particularly from Argo floats and Saildrones in the Southern Ocean^42^, can reduce air-sea CO_2_ flux uncertainties. While the zonal summertime sampling alone may not suffice to address seasonal biases, and substantial improvement in the Southern Ocean flux reconstructions is better achieved through year-round meridional sampling^42^, sailboats still contribute to an improved reconstruction of the air-sea CO_2_ fluxes in the Southern Ocean.

Rare underway pCO_2_ observations collected close to the frontal zones changed the air-sea CO_2_ flux estimate the most and can help to better understand these regions and their role in carbon uptake and the longer-term variation of the air-sea CO_2_ exchange. Compared to the Southern Ocean, races in the North Atlantic were less impactful due to the historical stronger observing network there. However, since the majority of races took place there, we still see sailboat pCO_2_ observations having a significant impact on regional air-sea CO_2_ flux densities. Thus, our analysis suggests that a declining number of observations in the North Atlantic as we currently see (www.socat.info), may lead to a significant impact on the global ocean carbon flux estimates^30^.

Added random measurement uncertainty (± 5 μatm) has a low impact on the overall air-sea CO_2_ flux estimate due to compensating errors. However, we illustrate the importance of including measurement uncertainty locally when investigating high-resolution fluxes. On the contrary, a potential measurement bias of 5 μatm results in a global flux bias larger than the detectable change due to 129 days of sailboat measurements. Although a measurement uncertainty of 5 µatm marks the lower end of achievable uncertainty ranges^43,44^, we show that even with this lower-end uncertainty fails to reveal any detectable impact when adding 129 days of *Seaexplorer* data. The impact of the measurement bias was more pronounced in the data-sparse Southern Ocean flux estimate, whereas the North Atlantic flux estimate proved to be more robust towards a measurement offset as a result of the denser existing measurement network^5^. Thus, our findings indicate that the quantity of the data has a greater influence on accuracy than the data quality in densely observed ocean areas.

We show the importance of cross-calibration and having a fleet simultaneously measuring pCO_2_ while closely sailing together. In this study, we detect any changes in the air-sea CO_2_ flux and attribute them to the integration of sailboat pCO_2_ observations. Although we show the difference induced by the *Seaexplorer* data, a conclusive answer to if, and to how much, the *Seaexplorer* data reduce the overall present-day uncertainty in the air-sea CO_2_ flux is still not provided. This should be explored in future studies. Considering the recurrence of sailboat races, they have the potential to improve reconstructive air-sea CO_2_ flux estimates on longer timescales in the future.

## Materials and methods

### Surface-ocean carbon dioxide data

Two sea surface carbon dioxide datasets were used in this study: (1) pCO_2_ measurements from underway shipboard and mooring data contained in the 1 × 1 degree gridded Surface Ocean CO_2_ Atlas SOCATv2022 from 1982 to 2021^5^ and (2) underway pCO_2_ measurements from the sailboat “Seaexplorer-Yacht Club de Monaco” (until 2019 “Malizia”) during offshore sailing and training events from 2018 to 2021. The former dataset includes the latter data as well, hence we artificially create a third dataset, where we exclude the *Seaexplorer* measurements from the SOCAT gridded dataset.

To quantify the changes in air-sea CO_2_ fluxes based on the addition of *Seaexplorer* data, we used these 2 datasets as starting points, i.e. (1) SOCATv2022 including *Seaexplorer* data (E1), and (2) SOCATv2022 excluding *Seaexplorer* data (E2). To assess the impact of a potential measurement accuracy of ± 5 μatm we created two more datasets by (3) adding a random uncertainty of ± 5 μatm to the *Seaexplorer* data (E3) (similar to differences observed during measurement campaigns^43,44^ where multiple pCO_2_ systems were compared to the membrane system used on sailboats) and by (4) adding a constant measurement offset of 5 μatm to the *Seaexplorer* data (E4), which is in theory possible but less likely considering the prescribed daily two-point calibration. The 5 µatm are based on the expected accuracy of flag C data in SOCAT, however, larger differences with the same systems have also been observed in field studies^43,44^. The system used here is configured with pressure measurements in the gas phase of the equilbration and makes daily zero and span gas calibrations. For a more detailed description of the measurements and the instrument setup and justification of the 5 µatm offset, we refer to^27^.

### pCO_2_ mapping and air-sea CO_2_ flux calculation

Mapped estimates of the sea surface pCO_2_ were created by applying the SOM-FFN method to all four datasets. Here we provide a brief overview of the method, whereas a more detailed description including evaluation can be found in^29,45^.

In the first step, a self-organizing map (SOM) clusters the ocean into 16 biogeochemical provinces based on common patterns in predictor variables. We used sea-surface temperature (SST) data^46^ (https://psl.noaa.gov/data/gridded/data.noaa.oisst.v2.html), sea-surface salinity (SSS) data^47^ (https://www.metoffice.gov.uk/hadobs/en4/—Analyses with Gouretski and Reseghetti (2010) bias corrections applied), a mixed layer depth (MLD) climatology^48^ (https://cerweb.ifremer.fr/deboyer/mld/Surface_Mixed_Layer_Depth.php), and a pCO_2_ climatology^49^ (https://www.ncei.noaa.gov/access/ocean-carbon-acidification-data-system/oceans/LDEO_Underway_Database/sumflux_2006c.txt) as predictors. In the second step, a feed-forward neural network (FFN) establishes non-linear relationships between the predictors and pCO_2_ observations within each province separately. It uses these relationships to reconstruct the missing pCO_2_ values within each province. The predictors for the FFN were SST, SSS, the MLD climatology as well as chlorophyll-a (http://www.globcolour.info; parameter CHL1 with the GSM L3 merging method), and the atmospheric CO_2_ concentration^50^ (https://gml.noaa.gov/ccgg/mbl/data.php). Prior to 1997, we used a monthly climatology from 1998 to 2002 for chlorophyll-a, given that chlorophyll-a became available only after the launch of satellites in 1997. The data for the FFN are divided into a training dataset to train on and a validation dataset used for validation within the method^29^.

From the four reconstructed pCO_2_ maps described above we estimate the air-sea CO_2_ flux based on a bulk gas transfer formulation with a quadratic relationship between windspeed and transfer velocity^29,51^ where we scale the mean gas transfer to a global average rate of 16.5 cm hr^−1^^52^. We calculate the difference between the flux estimate based on SOCAT with and without *Seaexplorer* data (E1 vs. E2) to quantify the impact on the air-sea CO_2_ flux. We further calculate the difference between the flux estimate based on *Seaexplorer* data with and without added measurement uncertainties (E1 vs. E3 and E4) to assess the impact of the expected measurement accuracy on the air-sea CO_2_ flux.

### Signal-to-noise-detection

To detect statistically significant differences and reduce the impact of random errors arising from methodological choices, we use a Monte Carlo approach to reconstruct and calculate each of the air-sea CO_2_ flux estimates (i.e. with *Seaexplorer* data, without *Seaexplorer* data, random error and constant offset—see above) 40 times with a varying split between the training and validation dataset to create for ensembles, i.e. ensemble E1 = *Seaexplorer* data, E2 = *Seaexplorer* data excluded, E3 = random measurement uncertainty, and E4 = fixed measurement bias. We gradually increased the number of runs and based on trial and error we found that the absolute difference between the two means of the ensembles between runs is nearly constant for 40 runs (Supplementary Fig. 3). To ensure the statistical significance of our results, we conducted a two-sample t-test and adjusted the resulting p-values in order to control the False Discovery Rate, i.e. the expected proportion of false discoveries among all significant results, to 5%^31^. The signal corresponds to adjusted p-values below 5%, indicating significance, while non-significant differences represent noise (Supplementary Fig. 4). The noise level is highest at the beginning of the time series as SOCAT contains few observations before 1990^5^, whereas the signal increases after 2016 as new *Seaexplorer* data made a difference (Supplementary Fig. 4).

### Regional focus

Finally, we set our focus on two main regions of interest, i.e. the North Atlantic, where most sailboat races took place, and on the Southern Ocean, where the longest race, the Antarctic circumnavigation race, took place. Furthermore,^53^ has highlighted significant uncertainties in the air-sea CO_2_ flux in both regions. We focused on three zonal bands in the Southern Ocean: the Polar Front, the Subantarctic Front, and the Northern Boundary^54^ including the respective areas within a 2-degree (or roughly 200 km) radius. We utilized the zonal bands enclosing the fronts as geographical reference points only to delineate zones in the Southern Ocean and to attribute differences caused by the addition of *Seaexplorer* data to these zones. Note that overlap between the frontal regions occurs. In the North Atlantic, we define the region as the area between 70°N, 0°, 85°W, and 20°E. The extent of the Southern Ocean is defined by south of 35°S.

To determine the data availability per region, we calculate the percentage of 1 × 1° pixels that were filled with *Seaexplorer* data at least once, regardless of the monthly availability.

### Supplementary Information

Supplementary Information.

## Data Availability

All data used and discussed in this article are freely available via www.socat.info. The datasets generated and/or analysed during the current study are available in the Zenodo repository, 10.5281/zenodo.10036579.
