# Supplementary material for: A detectable change in the air-sea CO2 flux estimate from sailboat measurements
Source: Sci Rep. 2024 Feb 9;14:3345. doi: 10.1038/s41598-024-53159-0 (PMC10858044; doi:10.1038/s41598-024-53159-0)
Supplement: Supplementary file 1 — Supplementary Information. [file 41598_2024_53159_MOESM1_ESM.docx]

# Supplementary Material

*
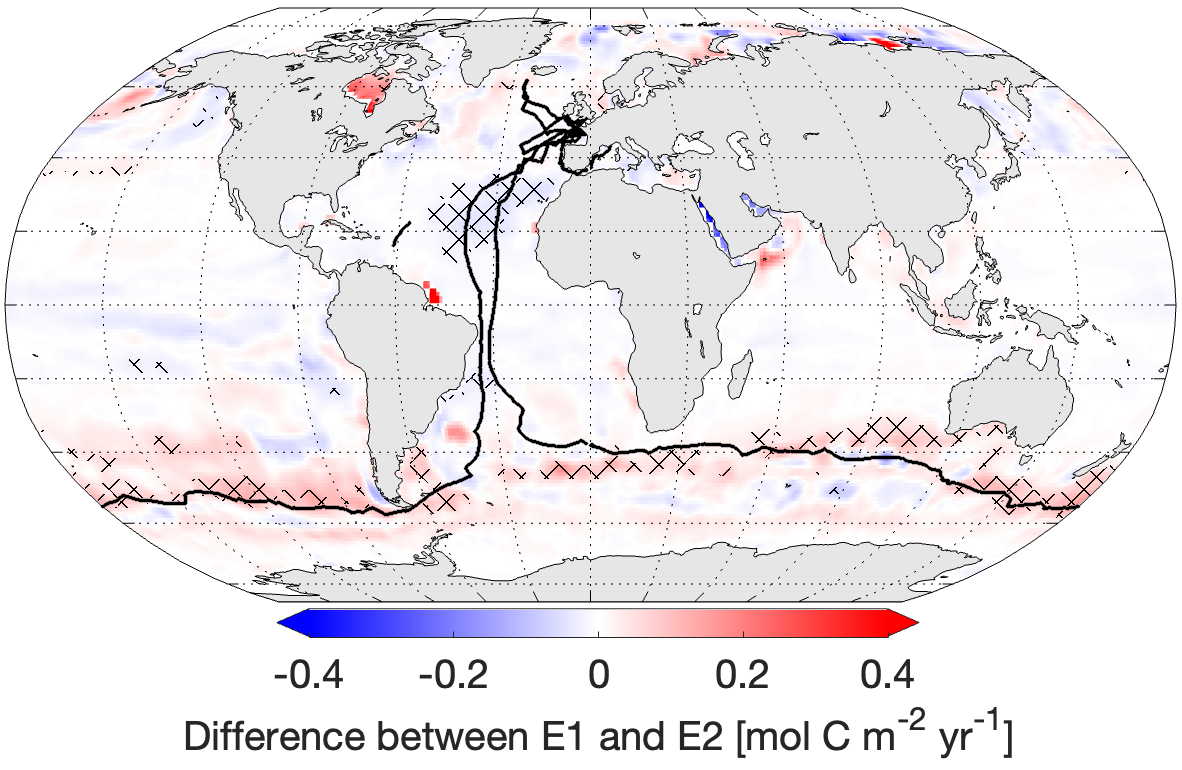
*

*Figure 1:* Difference between the air-sea CO₂ fluxes in ensemble E1 (based on SOCATv2022 including Seaexplorer data) and in ensemble E2 (based on SOCATv2022 excluding Seaexplorer data) averaged over 2018 - 2021*. Hatching indicates significant differences.* *Blue indicates increased carbon uptake due to the addition of Seaexplorer data, red indicates reduced carbon uptake due to the addition of Seaexplorer data. Black lines represent sailboat tracks from 2018 - 2021. Figure generated using a mapping package for MATLAB*^32^*.*

*
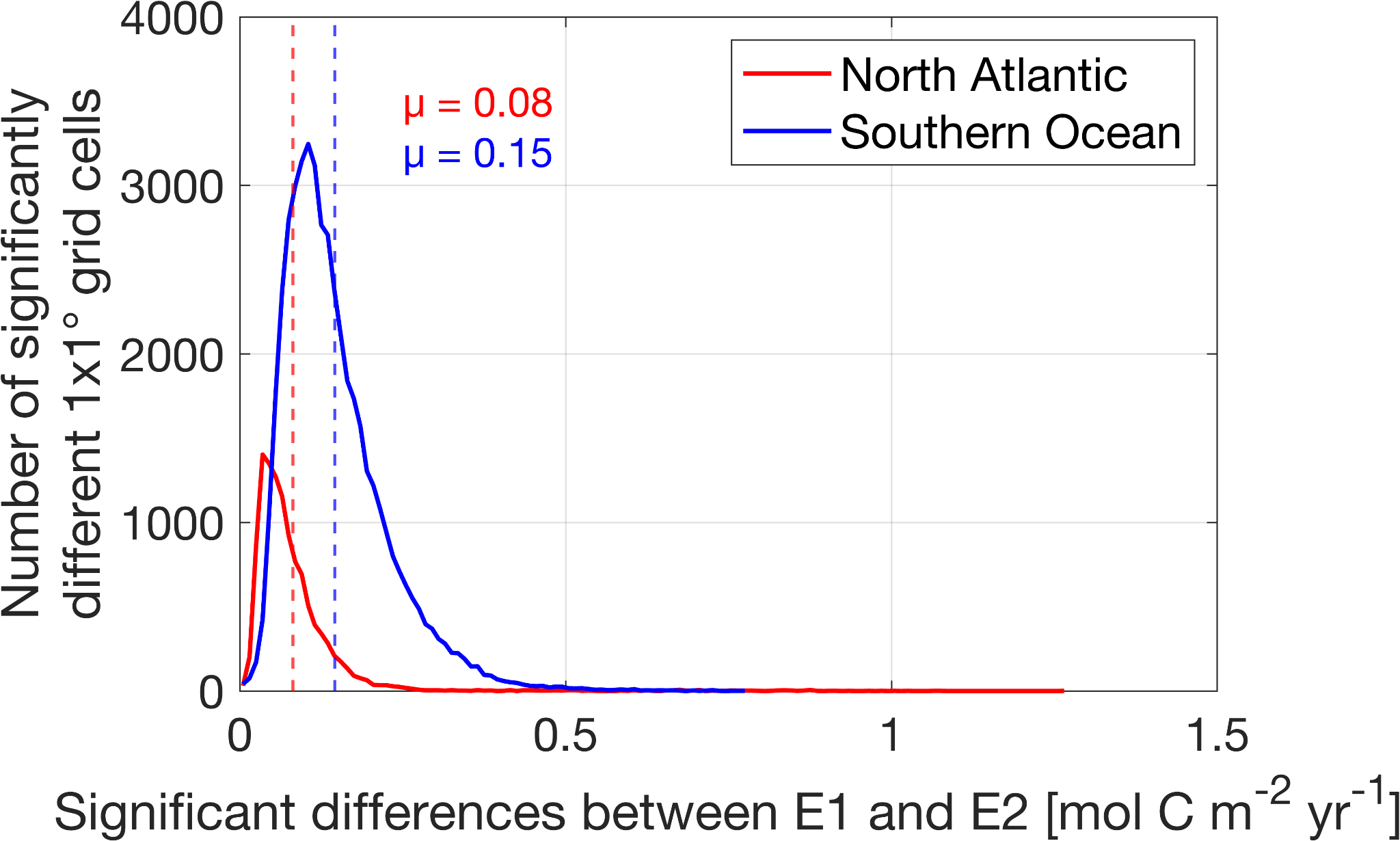
*

*Figure 2: Histogram of the absolute magnitude of significant differences between the air-sea CO_2_ flux E1 (based on SOCATv2022 including Seaexplorer data) and the air-sea CO_2_ flux E2 (based on SOCATv2022 excluding Seaexplorer data) in the Southern Ocean and the North Atlantic.*

*
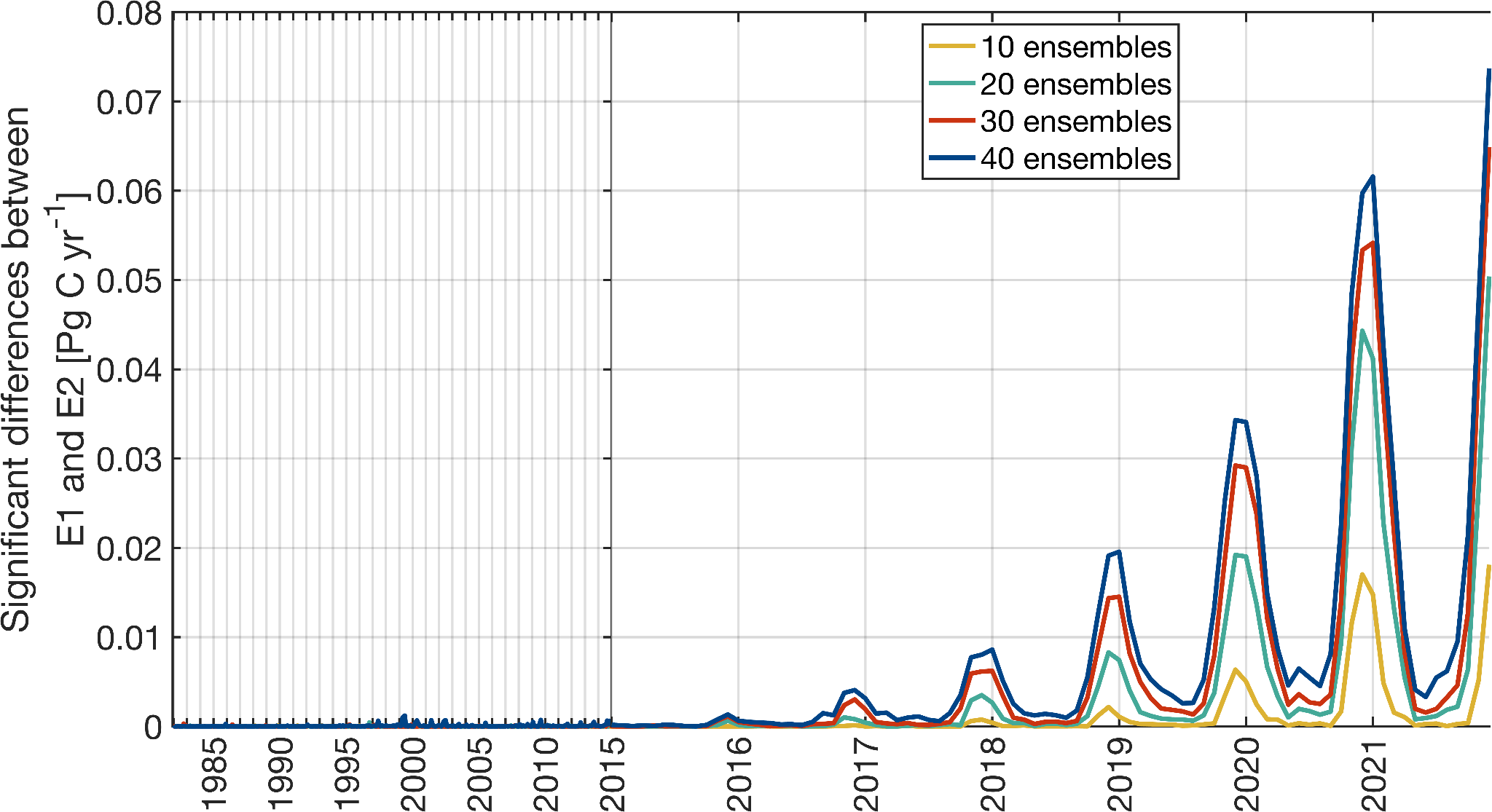
*

*Figure 3: Time series of the magnitude of significant differences between the air-sea CO₂ fluxes E1 and E2 (including and excluding sailboat pCO₂ data) based on the ensemble size of flux reconstructions. Changes in the magnitude of significant differences decrease with an increasing number of flux reconstructions as the random uncertainty is less well constrained.*

*Tab. 1: Integrated flux bias in Pg C yr⁻¹ in 2021*

|  | Due to missing Seaexplorer data  (comparison E1 and E2) | Due to random measurement uncertainty  (comparison E1 and E3) | Due to fixed measurement bias  (comparison E1 and E4) |
| --- | --- | --- | --- |
| Global | 0.04 | 0.01 | - 0.06 |
| North Atlantic | 0.00 | 0.00 | - 0.01 |
| Southern Ocean | 0.05 | 0.02 | - 0.03 |


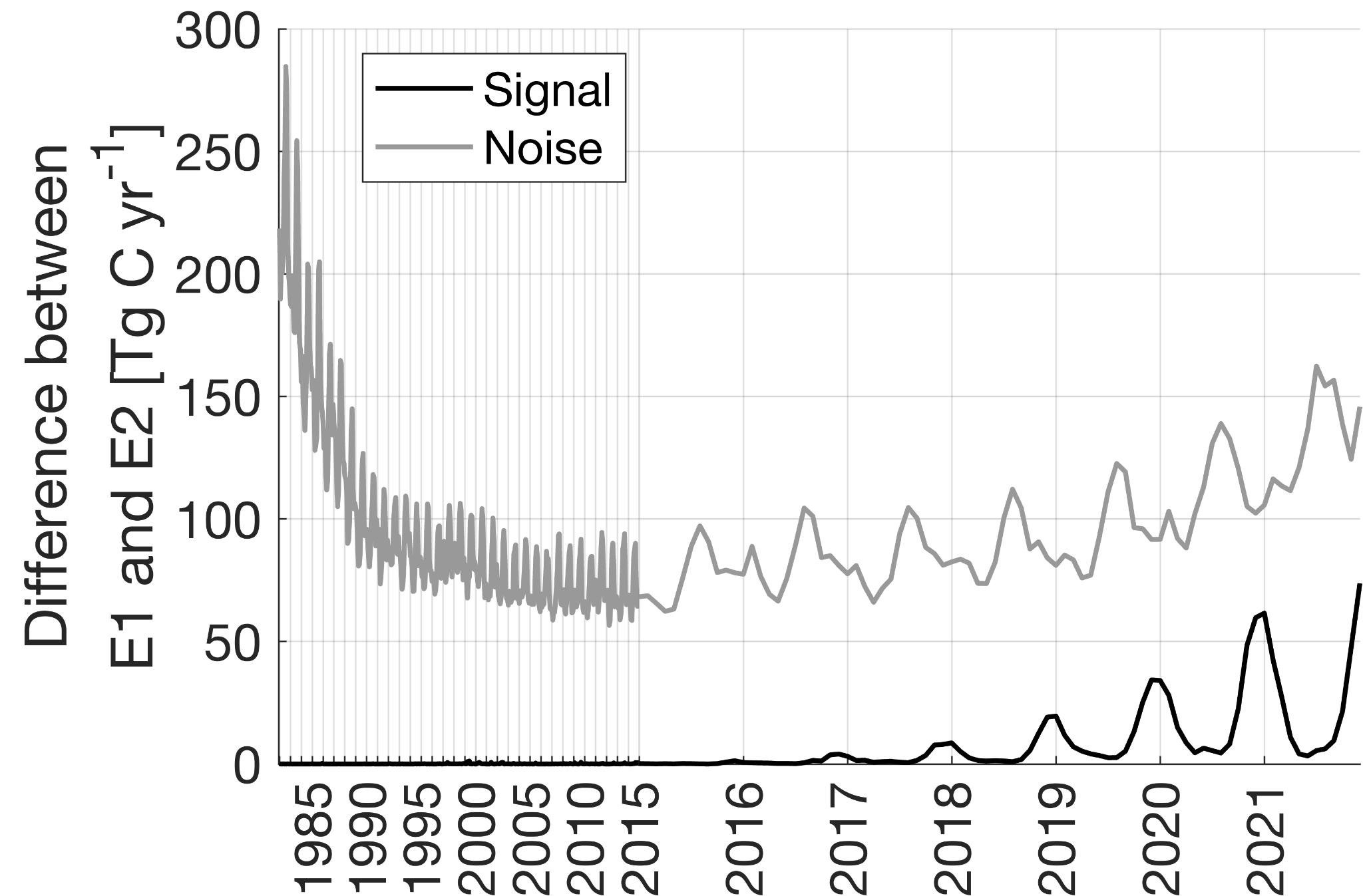


*Figure 4: Temporal development of signal and noise.*
